# 利妥昔单抗联合高强度化疗治疗散发性成人伯基特淋巴瘤的疗效及预后分析

**DOI:** 10.3760/cma.j.cn121090-20241130-00505

**Published:** 2025-02

**Authors:** 长明 董, 鹤松 邹, 文 张, 薇 刘, 轶 王, 慧敏 刘, 婷 谢, 恒 李, 齐 王, 文阳 黄, 树华 易, 刚 安, 录贵 邱, 德慧 邹

**Affiliations:** 1 中国医学科学院血液病医院（中国医学科学院血液学研究所），血液与健康全国重点实验室，国家血液系统疾病临床医学研究中心，细胞生态海河实验室，天津 300020 State Key Laboratory of Experimental Hematology, National Clinical Research Center for Blood Diseases, Haihe Laboratory of Cell Ecosystem, Institute of Hematology & Blood Diseases Hospital, Chinese Academy of Medical Sciences &Peking Union Medical College, Tianjin 300020, China; 2 天津医学健康研究院，天津 301600 Tianjin Institutes of Health Science, Tianjin 301600, China

**Keywords:** 伯基特淋巴瘤, 强烈免疫化疗方案, 疗效, 预后因素, Burkitt lymphoma, Intensive immunochemotherapy regimens, Efficacy, Prognostic factors

## Abstract

**目的:**

探讨利妥昔单抗联合高强度化疗治疗散发性成人伯基特淋巴瘤（BL）的治疗效果及预后因素。

**方法:**

回顾性分析2011年6月至2023年2月在中国医学科学院血液病医院诊治的30例初诊BL患者的临床和生存信息，采用Kaplan-Meier法计算生存率，运用Log-rank检验对预后因素进行单因素分析。

**结果:**

30例患者的中位年龄为43（24～66）岁，男女比例为3∶2。80.0％患者伴结外侵犯、53.3％伴骨髓侵犯、10.0％伴中枢神经系统侵犯，Ann Arbor分期Ⅲ～Ⅳ期占86.7％。伯基特淋巴瘤国际预后指数（BL-IPI）评分为低危（0分）、中危（1分）和高危（≥2分）者分别占比20.0％、43.3％和36.7％。所有患者均接受利妥昔单抗联合高强度化疗作为初始诱导治疗方案，客观缓解率和完全缓解率分别为80.0％和76.7％。中位随访时间为49（6～153）个月，5年的无进展生存（PFS）率和总生存（OS）率均为（76.7±7.7）％。4例局限期患者均获得持续的完全缓解（CCR）。10例对诱导治疗敏感的高危、进展期患者序贯一线auto-HSCT巩固治疗，除1例伴中枢神经系统侵犯的患者发生早期疾病进展而死亡，其余9例均获得CCR。BL-IPI低危、中危和高危组患者的5年PFS率分别为（83.3±15.2）％、100.0％和（45.5±15.0）％（*P*＝0.0069），OS率分别为（83.3±15.2）％、100.0％和（45.5±15.0）％（*P*＝0.0075）。诱导治疗期间的主要不良反应为骨髓抑制和继发感染，但均可对症处理。单因素分析显示，结外侵犯部位≥2个（*HR*＝4.90，95％ *CI* 1.02～23.45，*P*＝0.0329）、BL-IPI评分≥2分（*HR*＝12.62，95％ *CI* 2.59～61.62，*P*＝0.0021）和诱导治疗未达首次完全缓解（CR1）（*HR*＝31.86，95％ *CI* 4.19～242.20，*P*<0.0001）与更短的PFS期相关。

**结论:**

成人BL具有高度侵袭性，强烈免疫化疗方案整体上具有良好的疗效和安全性。局限期患者疗效佳，BL-IPI高危患者预后欠佳。诱导治疗敏感的高危、进展期患者序贯一线auto-HSCT巩固治疗可能进一步改善疗效。BL-IPI评分≥2分、结外侵犯部位≥2个和诱导治疗未达CR1是成人BL患者的预后不良因素。

## Abstract (en)

**Objective:**

To explore the therapeutic efficacy and prognostic factors of combined rituximab and intensive chemotherapy for sporadic adult Burkitt lymphoma（BL）.

**Methods:**

This retrospective study examined the clinical and survival data of 30 patients newly diagnosed with BL between July 2011 and February 2023 at the Blood Diseases Hospital. Kaplan-Meier method was used for survival analysis, and the log-rank test was used for univariate analysis of prognostic factors.

**Results:**

The median age of the 30 patients was 43 years（24 – 66 years）, and the male to female ratio was 3:2. Extranodal invasion was present in 80％ of the patients, with involvement of the bone marrow in 53.3％ and central nervous system in 10.0％. The Ann Arbor stage was Ⅲ and Ⅳ in 86.7％. According to the number of Burkitt Lymphoma International Prognostic Index（BL-IPI）risk factors, patients were classified as low risk（0）in 20.0％, intermediate risk（1）in 43.3％, and high risk（≥2）in 36.7％. All patients were treated with an induction regimen of rituximab combined with intensive chemotherapy, with objective and complete response rates of 80.0％ and 76.7％, respectively. The median follow-up was 49 months（6–153 months）, and the 5-year progression-free survival（PFS）and overall survival（OS）rates were both（76.7±7.7）％. All patients with limited stage（*n*＝4）achieved continuous complete remission（CCR）. Patients who had high risk, advanced stage sensitive to induction therapy（*n*＝10）sequentially received first-line autologous hematopoietic stem cell transplantation（auto-HSCT）as consolidation therapy; 9 patients achieved CCR, whereas 1 patient with central nervous system invasion developed early disease progression and died. The BL-IPI low, intermediate, and high risk groups had respective 5-year PFS rates of（83.3±15.2）％, 100.0％, and（45.5±15.0）％（*P*＝0.0069）and OS rates of（83.3±15.2）％, 100.0％, and（45.5±15.0）％（*P*＝0.0075）. The main adverse effects of induction therapy were myelosuppression and secondary infections, which were effectively managed by appropriate symptomatic treatment. Univariate analysis demonstrated that worse PFS was associated with BL-IPI score ≥2（*HR*＝4.90, 95％ *CI* 1.02–23.45, *P*＝0.0329）; extranodal invasion at ≥2 sites（*HR*＝12.62, 95％ *CI* 2.59–61.62, *P*＝0.0021）; and failure to achieve first complete response（CR1）after induction therapy（*HR*＝31.86, 95％ *CI* 4.19–242.20, *P*<0.0001）.

**Conclusion:**

Intensive immunochemotherapy regimens were effective and well-tolerated by adult patients with highly aggressive BL. Treatment efficacy was ideal in patients with limited-stage disease, whereas prognosis was unsatisfactory in patients with high-risk BL-IPI. Sequential first-line auto-HSCT consolidation therapy may further improve outcomes in patients with high-risk advanced-stage disease who are sensitive to induction therapy. BL-IPI score ≥2, extranodal invasion at ≥2 sites, and failure to achieve CR1 after induction therapy were adverse prognostic factors in adult patients with BL.

伯基特淋巴瘤（BL）作为一种高度侵袭性成熟B细胞淋巴瘤，常见于儿童和青少年，但仅占成人非霍奇金淋巴瘤（NHL）的1％～2％[1]–[2]。依据流行病学与临床特征的不同，世界卫生组织（WHO）血液及淋巴系统肿瘤分类将BL分为3个亚型：地方性、散发性和免疫缺陷相关性BL[3]。BL具有显著的临床表现，即迅速的淋巴瘤生长和结外侵犯，包括胃肠道、骨髓和中枢神经系统等，若不及时治疗，患者可在数月内死亡[1]。我国的BL患者绝大多数为散发性，在成年人中发病率低，相关的系统性报道较少。本研究就本中心收治的30例初诊BL患者的临床和生存资料进行汇总分析，旨在探讨我国成人BL的临床特征、治疗方案的安全性与有效性以及预后相关因素。

## 病例与方法

1. 病例资料：纳入2011年6月至2023年2月就诊于中国医学科学院血液病医院的30例初诊BL患者。纳入标准：①年龄≥18岁。②符合2022版WHO血液及淋巴系统肿瘤分类中BL的病理诊断标准[3]：细胞形态学上表现为单一、中等大小的肿瘤细胞弥漫浸润，细胞核中等大小、居中、嗜碱性，胞质呈强嗜碱性，多伴有脂质空泡，且常见“星空现象”；免疫学上表现为成熟的B细胞表型：CD19、CD20、CD79A和PAX5阳性，生发中心标志CD10和BCL6阳性，CD5、BCL2、TdT阴性，Ki-67指数>90％或接近100％；分子遗传学上具有典型的MYC基因易位而无BCL2和（或）BCL6易位。③初次就诊于我院且接受至少3个疗程的一线利妥昔单抗联合高强度化疗方案治疗，序贯或不序贯一线auto-HSCT巩固治疗。④HIV检测阴性。排除标准：①年龄<18岁；②既往外院治疗后的复发/难治患者。本研究通过中国医学科学院血液病医院伦理委员会审批（批件号：IIT2021030-EC-1）。

2. 治疗方案：所有患者均接受利妥昔单抗联合高强度化疗作为初始诱导治疗，序贯或不序贯auto-HSCT治疗作为一线治疗方案。初始诱导治疗方案包括：R-HyperCVAD/R-MA方案（13例）、R-CODOX-M/R-IVAC方案（6例）、R-DA-EDOCH方案（6例）和R-COAMTD/R-VCAMD方案（5例）。上述方案的具体用法及剂量见文献[4]–[8]。对于诱导治疗敏感［4个疗程获得完全缓解（CR）或部分缓解（PR）］的高危、进展期患者，根据患者年龄及意愿选择序贯auto-HSCT治疗或完成6～8个疗程诱导治疗。所有患者均接受甲氨蝶呤联合阿糖胞苷以及地塞米松三联鞘内注射以防治淋巴瘤的中枢神经系统侵犯，中位鞘内注射的次数为7（3～13）次。

3. 疗效评估和不良反应分级：根据2014年Lugano疗效评价标准[9]，采用PET-CT和（或）CT，初诊时合并骨髓侵犯的患者需同步复查骨髓流式细胞术和病理免疫组化进行疗效评估。疗效评估的时机包括治疗中期（2～4个疗程后）、治疗结束后和随访期。治疗效果分为CR、PR、疾病稳定（SD）和疾病进展（PD）。客观缓解率（ORR）为CR率和PR率之和。化疗相关不良反应依据CTCAE分级v5.0进行评估。

4. 随访：通过查阅病历和电话进行随访，随访截止日期为2024年10月20日。无进展生存（PFS）期定义为从接受治疗至首次出现疾病进展、或因任何原因死亡或末次随访的时间。总生存（OS）期定义为自明确诊断至任何原因导致死亡或末次随访的时间。

5. 统计学分析：使用Graphpad prism 10.1和SPSS 27.0统计软件对临床数据进行统计学处理。计量资料以中位数（范围）或*x*±*s*来描述，计数资料以例数（百分比）来描述。采用Kaplan-Meier法计算PFS和OS并绘制生存曲线。预后的单因素分析采用Log-rank检验；多因素分析采用Cox回归分析。以*P*<0.05（双侧）为差异具有统计学意义。

## 结果

1. 临床特征：如表1所示，30例患者的中位年龄为43（24～66）岁，男女比例为3∶2。11例（36.7％）患者伴有B症状、19例（63.3％）LDH水平升高。24例（80.0％）初诊时伴有结外侵犯、16例（53.3％）骨髓侵犯、3例（10.0％）中枢神经系统侵犯。Ann Arbor分期Ⅰ～Ⅱ期（局限期）和Ann Arbor分期Ⅲ～Ⅳ期（进展期）的患者分别为4例（13.3％）和26例（86.7％）。伯基特淋巴瘤国际预后指数（BL-IPI）评分为低危（0分）、中危（1分）和高危（≥2分）的患者分别为6例（20.0％）、13例（43.3％）和11例（36.7％）。

**表1:** 30例伯基特淋巴瘤患者的临床特征

临床特征	例（％）
年龄	
<40岁	14（46.7）
≥40岁	16（53.3）
性别	
女	12（40.0）
男	18（60.0）
B症状	
无	19（63.3）
有	11（36.7）
ECOG评分	
0～1分	25（83.3）
≥2分	5（16.7）
LDH	
正常	11（36.7）
1～3倍ULN	4（13.3）
>3倍ULN	15（50.0）
骨髓侵犯	
无	14（46.7）
有	16（53.3）
中枢神经系统侵犯	
无	27（90.0）
有	3（10.0）
结外侵犯部位	
0个	6（20.0）
1个	13（43.3）
≥2个	11（36.7）
Ann Arbor分期	
Ⅰ～Ⅱ期	4（13.3）
Ⅲ～Ⅳ期	26（86.7）
BL-IPI评分	
0分	6（20.0）
1分	13（43.3）
≥2分	11（36.7）

**注** ECOG：美国东部肿瘤协作组；LDH：乳酸脱氢酶；ULN：正常值上限；BL-IPI：伯基特淋巴瘤国际预后指数

2. 治疗效果及生存分析：中位的一线诱导治疗疗程为6（3～8）个，30例患者初始诱导治疗后的ORR为80.0％、CR率为76.7％，6例（20.0％）发生PD。中位随访时间49（6～153）个月，中位OS期和PFS期均未达到，预期5年PFS率和OS率均为（76.7±7.7）％。

4例局限期患者中2例接受R-CODOX-M/R-IVAC方案治疗，1例接受R-DA-EDOCH方案治疗，1例接受R-HyperCVAD/R-MA方案治疗，诱导治疗后的CR率为100％，且该4例患者截至随访日期均为持续的CR（CCR），时间分别为38个月、61个月、65个月和101个月。

26例进展期患者的诱导治疗方案包括：R-HyperCVAD/R-MA方案（12例）、R-CODOX-M/R-IVAC方案（4例）、R-DA-EDOCH方案（5例）和R-COAMTD/R-VCAMD方案（5例），其ORR和CR率分别为76.9％和73.1％，预期5年PFS率和OS率均为（73.1±8.7）％。10例对诱导治疗敏感（9例CR和1例PR）的高危患者序贯一线auto-HSCT巩固治疗，排除1例伴有中枢神经系统侵犯的患者因移植后发生早期PD而死亡，其余9例均获得CCR。19例诱导治疗获得CR的患者中，排除1例发生PD而死亡，其余18例均获得CCR；1例诱导治疗获得PR的患者，在序贯一线auto-HSCT巩固治疗后CCR；6例诱导治疗无效的患者虽经挽救治疗（3例挽救化疗、1例auto-HSCT、1例allo-HSCT和1例姑息治疗），但均死亡（5例死于PD，1例死于allo-HSCT相关感染），PD后的中位生存期仅为3（1～10）个月。

3. 预后分析：如表2所示，单因素分析显示，结外侵犯部位≥2个（*HR*＝4.90，95％ *CI* 1.02～23.45，*P*＝0.0329）、BL-IPI评分≥2分（*HR*＝12.62，95％ *CI* 2.59～61.62，*P*＝0.0021）和诱导治疗未达首次完全缓解（CR1）（*HR*＝31.86，95％ *CI* 4.19～242.20，*P*<0.0001）是影响PFS的预后不良因素。结外侵犯部位≥2个（*HR*＝5.39，95％ *CI* 1.10～26.51，*P*＝0.0219）、BL-IPI评分≥2分（*HR*＝12.38，95％ *CI* 2.56～20.08，*P*＝0.0024）和诱导治疗未达CR1（*HR*＝29.56，95％ *CI* 4.09～213.70，*P*<0.0001）是影响OS的预后不良因素。进一步多因素分析未显示独立的预后因素。

**表2:** 关于30例伯基特淋巴瘤患者的单因素生存分析

临床特征	无进展生存		总生存	
	*HR*（95％ *CI*）	*P*值	*HR*（95％ *CI*）	*P*值
年龄≥40岁	2.38（0.54～10.48）	0.2797	2.23（0.51～9.85）	0.3186
男性	1.62（0.36～7.38）	0.5563	1.81（0.40～8.11）	0.4683
B症状	2.26（0.49～10.47）	0.2672	2.48（0.52～11.73）	0.2133
ECOG评分≥2分	2.16（0.28～16.66）	0.3386	2.46（0.29～20.80）	0.2600
LDH>3倍ULN	2.58（0.59～11.37）	0.2335	2.49（0.57～10.94）	0.2544
骨髓侵犯	1.19（0.27～5.24）	0.0527	1.14（0.26～5.05）	0.8588
结外侵犯部位≥2个	4.90（1.02～23.45）	0.0329	5.39（1.10～26.51）	0.0219
Ann Arbor分期（Ⅲ～Ⅳ期）	3.32（0.40～27.48）	0.2659	3.31（0.40～27.34）	0.2660
BL-IPI评分≥2分	12.62（2.59～61.62）	0.0021	12.38（2.56～20.08）	0.0024
诱导治疗未达到CR1	31.86（4.19～242.20）	<0.0001	29.56（4.09～213.70）	<0.0001

**注** ECOG：美国东部肿瘤协作组；ULN：正常值上限；BL-IPI：伯基特淋巴瘤国际预后指数；CR1：首次完全缓解

4. BL-IPI评分的验证：低危组、中危组和高危组患者的5年PFS率分别为（83.3±15.2）％、100.0％和（45.5±15.0）％（*P*＝0.0069），OS率分别为（83.3±15.2）％、100.0％和（45.5±15.0）％（*P*＝0.0075）。低危组与中危组的5年PFS率和OS率差异均无统计学意义（均*P*>0.05）。高危组与低中危组的5年PFS率分别为（45.5±15.0）％和（94.7±5.1）％（*P*＝0.0021）、OS率分别为（45.5±15.0）％和（94.7±5.1）％（*P*＝0.0024），见图1。

**图1:**
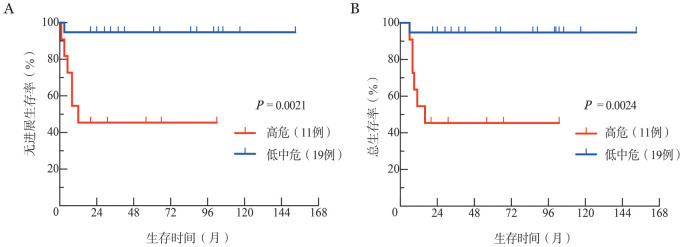
伯基特淋巴瘤国际预后指数高危和低中危的伯基特淋巴瘤患者的无进展生存曲线（A）和总生存曲线（B）

5. 安全性与耐受性分析：所有患者均在化疗期间出现3～4级的重度骨髓抑制，其中粒细胞缺乏性发热19例（63.3％）、血小板减少相关性出血4例（13.3％）。3～4级非血液学不良反应中，以口腔黏膜炎最为常见（26.7％）、其余常见的不良反应依次为肺部感染7例（23.3％）、周围神经病变4例（13.3％）、胃肠道感染3例（10.0％）、败血症3例（10.0％）、皮疹2例（6.7％）和肝功能损害1例（3.3％）。上述不良反应均可对症处理，无治疗相关死亡发生。5例（16.7％）因骨髓抑制期的继发感染（3例）或出血事件（2例）由R-HyperCVAD/R-MA方案或R-CODOX-M/R-IVAC方案更换为相对低剂量的R-DA-EDOCH方案；3例（10.0％）因骨髓抑制期的继发感染推迟化疗，中位推迟时间为28（14～28）d。

## 讨论

BL作为一种高度侵袭性的血液系统恶性肿瘤，在儿童与青少年中常见，但在成年人中发病率较低[1]。我国成人BL患者的亚型以散发性为主，由于其发病率较低，国内相关的大样本报道较少。

BL对NHL的传统化疗方案（如CHOP方案）的治疗反应差，治愈率不足30％[4]。但随着利妥昔单抗联合短周期、高强度化疗方案的应用，90％以上的儿童和青少年可得到治愈，65％～80％的成人可获得长期缓解[1]。Evens等[10]的多中心真实世界研究中，633例BL患者接受高强度化疗（90％联合应用利妥昔单抗），主要方案包括HyperCVAD/MA、CODOX-M/IVAC和DA-EPOCH方案，3年PFS率和OS率分别为65％和70％；且上述3种诱导治疗方案的疗效差异无统计学意义。国内一项中山大学肿瘤防治中心的单中心回顾性研究[11]中，采用调整的R-CODOX-M/IVAC方案治疗123例初治成人BL患者，ORR为87％，3年无事件生存（EFS）率和OS率分别为81.2％和92.1％。而本研究30例患者较上述研究起病时年龄偏高，伴有B症状、LDH水平升高、处于进展期以及伴有骨髓侵犯和中枢神经系统侵犯的患者比例均更高，表明本研究中的患者群体肿瘤负荷更高，具有更多高危因素。初始诱导治疗后的ORR和CR率分别为80.0％和76.7％，5年PFS率和OS率均为（76.7±7.7）％，与上述研究报道结果[10]–[11]类似，同样表明利妥昔单抗联合高强度化疗方案治疗成人BL有良好的疗效和安全性。

关于auto-HSCT是否应作为BL患者的一线巩固治疗方案目前仍无定论。在传统化疗时代，高强度化疗首次缓解后序贯auto-HSCT，患者可有生存获益，长期的PFS率可达到51％～72％[12]–[13]。但自进入免疫联合化疗时代以来，利妥昔单抗联合多药的高强度化疗方案已在BL患者中取得显著疗效，关于一线auto-HSCT巩固治疗能否使BL患者有生存获益尚待进一步研究和探讨。Maramattom等[14]的研究中显示CR1组序贯auto-HSCT的5年PFS率和OS率分别为78％和83％，非CR1组序贯auto-HSCT的5年PFS率和OS率分别为27％和31％。杨萍等[15]的研究中7例患者在CR1后序贯auto-HSCT作为巩固治疗，3年OS率为100％。本研究中10例对诱导治疗敏感的高危、进展期患者序贯一线auto-HSCT巩固治疗，排除1例伴中枢神经系统侵犯的患者在移植后因早期PD而死亡，其余9例均获得CCR。由以上多项研究可见，诱导化疗敏感序贯一线auto-HSCT巩固治疗可能进一步改善BL患者的长期生存，但对于具体哪一类患者群体能从中取得最佳的生存获益，仍需更大样本量的研究来探索。

关于预后评估方面，由于BL老年患者比例较低，且初诊时多处于进展期且伴LDH水平升高，IPI评分并不能很好地适用于BL患者的预后评估[16]。Olszewski等[17]的研究中提出根据BL-IPI评分进行预后分层：低危（0分）、中危（1分）和高危（≥2分）组的生存有显著差异，3年PFS率分别为92％、72％和53％（*P*<0.0001）、OS率分别为96％、76％和59％（*P*<0.0001）。本研究同样显示，高危患者的长期生存率显著低于低中危患者，而低危与中危患者的长期生存率差异无统计学意义，可能与样本量较小有关。Sýkorová等[16]的研究中多因素分析显示诱导治疗未达CR、伴有结外侵犯和Ann Arbor Ⅳ期与较短的PFS期相关，诱导治疗未达CR和伴中枢神经系统侵犯与较短的OS期相关。本研究单因素分析显示结外侵犯部位≥2个、BL-IPI≥2分和诱导治疗未达CR1与更短的PFS和OS期相关，但进一步多因素分析未显示独立的预后因素，可能与整体的样本量较小有关。以上结果再次验证了BL-IPI对于识别高危患者的价值，多个结外侵犯部位以及诱导治疗效果欠佳对于早期识别高危患者也有一定的指导意义。BL-IPI高危患者的治疗效果和生存仍欠佳，需进一步探索有效的整体治疗策略。

对于复发/难治BL患者，尚缺乏有效的挽救治疗措施，其生存率仅为10％～36％[18]。Maramattom等[14]的研究结果显示，接受auto-HSCT的复发/难治BL患者的5年PFS率和OS率分别为27％和31％，接受allo-HSCT的5年PFS率和OS率分别为19％和20％。Burkhardt等[19]的研究中，254例复发/难治儿童BL患者接受auto-HSCT和allo-HSCT的生存率分别为44％和46％，而未接受HSCT的生存率仅为3％。因此，对于复发/难治BL患者，仍需探索新的治疗手段，HSCT、分子靶向药物以及细胞免疫治疗的合理应用可能是未来有效的策略。

综上所述，本研究作为小样本量的回顾性研究，具有一定的局限性，但研究结果仍然表明我国成人BL存在侵袭性强的临床特征如疾病分期多为进展期以及结外侵犯常见等。在治疗上，利妥昔单抗联合高强度化疗整体上具有良好的疗效和安全性，局限期患者的疗效佳，BL-IPI高危患者的预后欠佳；诱导治疗敏感的高危、进展期患者序贯一线auto-HSCT巩固治疗可能进一步改善疗效。BL-IPI高危、多个结外侵犯部位和对诱导治疗不敏感对于早期识别高危患者具有指导意义；对于上述患者，疾病一旦进展，预后极差，尚需探索更有效的治疗策略。
